# Transcatheter Intervention Is Associated With Visceral Decongestion in Patients With Severe Tricuspid Regurgitation

**DOI:** 10.1002/clc.70497

**Published:** 2026-10-22

**Authors:** Franz Josef Putz, Johannes Spahn, Paul C. Kranert, Franziska Grewe, Moritz Haus, Michael Wester, Valeska Bienert, Maximilian Trum, Christian Schach, Andreas Luchner, Christoph Birner, Bernhard Unsöld, Miriam C. Banas, Bernhard Banas, Lars S. Maier, Kurt Debl, Michael Paulus, Christine Meindl

**Affiliations:** ^1^ Department of Nephrology University Hospital Regensburg Regensburg Germany; ^2^ Department of Internal Medicine II University Hospital Regensburg Regensburg Germany; ^3^ Department of Cardiology Krankenhaus Barmherzige Brüder Regensburg Germany; ^4^ Department of Internal Medicine I Klinikum St. Marien Amberg Germany; ^5^ Department of Internal Medicine I University Hospital Gießen and Marburg Marburg Germany

**Keywords:** renal vein flow, tricuspid regurgitation, TTVI, ultrasound, visceral congestion

## Abstract

**Introduction:**

Tricuspid regurgitation (TR) is frequently associated with severe venous congestion, increasingly recognized as a key factor in the development of cardiorenal syndrome. While transcatheter tricuspid valve intervention (TTVI) has shown to be effective in TR reduction, their impact on visceral venous congestion remains unknown. The aim of our study was to investigate visceral venous congestion in patients with severe TR undergoing TTVI.

**Methods:**

We prospectively enrolled 30 patients undergoing TTVI for severe TR. Renal venous blood flow was assessed by Doppler ultrasound examination at baseline and 1 to 3 months after the procedure. A 5‐grade rating scale was developed to categorize venous flow from continuous (Grade I°) through discontinuous to a pendulous venous flow (Grade V°) within the kidneys.

**Results:**

Transcatheter edge‐to‐edge repair (TEER, *n* = 24) was performed in 24 patients; 6 patients underwent heterotopic caval valve implantation. At baseline, severe renal congestion was present in most participants, with 20 patients showing venous flow profiles IV or V. Venous flow profiles strongly correlated with invasively measured right atrial mean pressure and V wave (*p* < 0.01). At follow‐up, venous renal blood flow profiles improved significantly (*p* = 0.003), reducing patients with profiles IV or V from 20 to 9. Portal vein flow showed comparable decongestive changes. In TEER patients, reduction of TR ≥ 2 grades was associated with improved renal vein profile at follow‐up (*p* = 0.002).

**Conclusion:**

Successful TTVI leads to visceral decongestion. Renal venous flow currently represents a physiological marker of congestion that may complement conventional echocardiographic parameters; its role in guiding patient selection or timing of intervention remains to be determined in larger studies.

## Practitioner Points

- In this prospective pilot study, visceral venous congestion was systematically assessed by ultrasound in patients undergoing TEER and heterotopic caval valve implantation for severe TR.
- Successful TEER was associated with a marked reduction in sonographic venous congestion and concomitant improvement in functional capacity, health‐related quality of life, and loop diuretic dose.
- The proposed ultrasound framework may help to characterize the systemic impact of TR and its interventional treatment; its potential role in refining patient selection and follow‐up strategies warrants investigation in larger, prognostic studies.

## Graphical abstract

Patients with severe symptomatic tricuspid regurgitation were assigned to transcatheter tricuspid valve intervention. Most patients showed discontinuous renal vein blood flow (arrows, Grade IV°) prior to TTVI (baseline). At follow‐up after 1 to 3 months, patients showed continuous renal vein blood flow profile (arrows, Grade I°) representing a marked visceral decongestion.

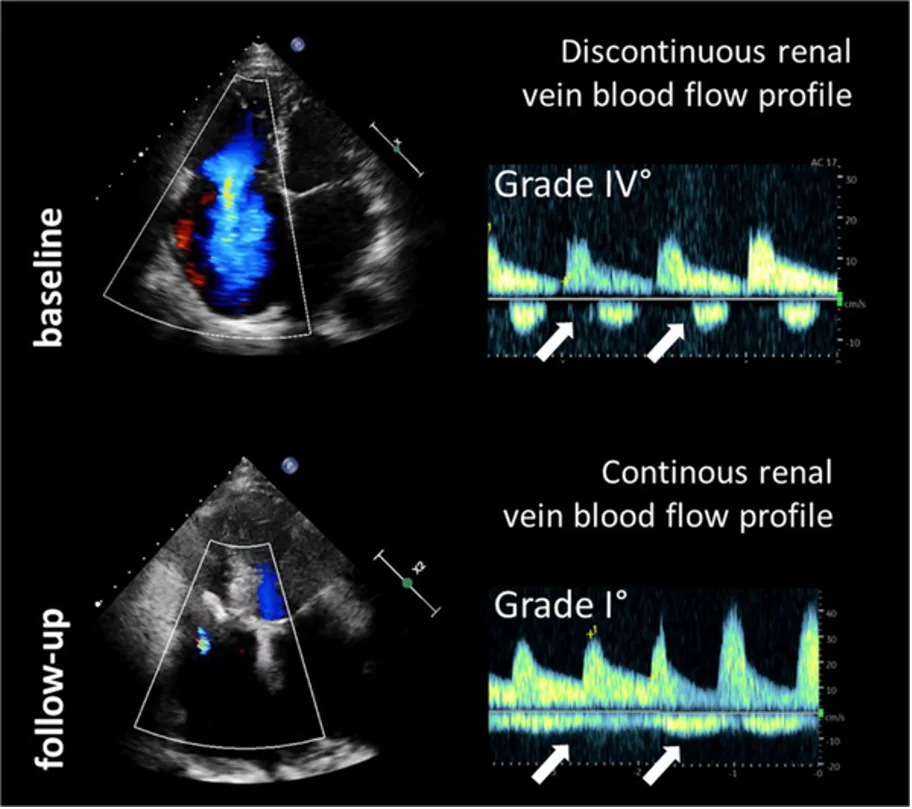

**Abbreviations**
HCVR: heterotopic caval valve replacement
RVFP: renal vein flow profile
TEER: transcatheter edge‐to‐edge repair
TR: tricuspid regurgitation
TTVI: transcatheter tricuspid valve intervention
VFI: venous flow index
VSI: venous stasis index
VVC: visceral venous congestion

## 1 Introduction

Tricuspid valve regurgitation (TR) is accompanied by high rates of morbidity, mortality, and hospitalizations for heart failure [1, 2, 3]. Severe TR is often diagnosed at a late stage when signs and symptoms of right‐sided heart failure (RHF) are advanced. Severe TR is characterized by increased venous pressures, volume overload, and a reduction of stroke volume, causing cardiorenal syndrome [4]. At that time, management is challenging: isolated surgical repair is associated with high procedural risk, while contemporary transcatheter strategies including TEER, transcatheter tricuspid valve replacement, and heterotopic caval valve implantation offer therapeutic options that must be tailored to the individual anatomy, comorbidities, and clinical presentation [5, 6, 7]. Transcatheter tricuspid valve interventions (TTVI) have shown to be effective in TR reduction and symptom improvement [8]. However, both patient selection and timing of the intervention to maximize clinical benefit remain challenging [9].

Severe TR results in diminished forward cardiac output and increased right‐sided systemic venous pressure. Consequently, volume overload appears, resulting in visceral congestion and organ dysfunction [10, 11, 12, 13]. The TRILUMINATE Pivotal trial investigated end‐organ function in patients with TR undergoing transcatheter edge‐to‐edge repair (TEER) [14]. In patients with moderate to severe dysfunction of the kidney as well as of the liver at baseline hospitalization for heart failure, death occurred more often within 12 months irrespective of treatment. Estimated glomerular filtration rate (eGFR) did not improve significantly in the TEER group compared to the control group. However, parameters of kidney and liver function ameliorated after successful TEER in a subgroup analysis [14].

Ultrasound is increasingly recognized as a method for identifying and quantifying visceral venous congestion (VVC) and end‐organ damage. The quantification is based on duplex sonographic flow profiles in the kidneys and the liver. There is evidence that ultrasound‐based grading systems have prognostic value for acute kidney injury after cardiac surgery [15]. In patients with acute heart failure severe VVC was associated with elevated left ventricular filling pressures, an impaired right atrial and right ventricular function and predicted in‐hospital mortality in these patients [16]. Outpatients with heart failure benefit of multi‐organ ultrasound to detect VVC [17]. Intrarenal venous flow profiles (RVFP) changing from continuous to discontinuous flow pattern were concomitant with higher right atrial pressures and also worse prognosis in patients with acute and chronic heart failure [17, 18].

The impact of TTVI on VVC and its role in preventing or ameliorating cardiorenal syndrome remains unknown. Therefore, the aim of our study was to investigate renal venous flow and portal vein flow profiles in patients with severe TR undergoing TTVI.

## 2 Methods

### 2.1 Study Design

From September 2022 to August 2024, 30 patients with symptomatic severe TR were prospectively enrolled in the single‐center RETORT‐TR trial (Regensburg Trial on TTVR Techniques in TR). The investigation conforms with the principles outlined in the Declaration of Helsinki. All included patients gave informed consent before enrollment, and the trial was approved by the local ethics committee (21‐2364‐101). No external funding was obtained. Of all participants, clinical data and medication were collected, and serial blood and urine sampling was performed 24 h before and 4 weeks after the procedure.

Changes in loop diuretic therapy during follow‐up were not protocolized but reflected routine clinical decision‐making based on symptoms, physical examination and ultrasound. Diuretic dose at baseline and follow‐up was systematically recorded and analyzed. However, medication adjustment may itself influence venous congestion and volume status and therefore represents a potential source of confounding in the interpretation of RVFP and VFI changes.

In addition, transthoracic (TTE) and transesophageal (TOE) echocardiography as well as ultrasound of the abdomen were conducted. 6‐min walk tests and the EQ‐5D‐5L questionnaire were performed to investigate patients' physical and mental status. Invasive right heart catheterization was conducted according to current standards before the procedure.

Two‐dimensional TTE echocardiography was performed using Epiq CVx and Epiq CVxi ultrasound systems with S5‐1 transducers (Philips Medical Systems, Amsterdam, The Netherlands). Ultrasound evaluation was conducted using the LOGIQ E10 ultrasound system with a C1‐6 transducer (GE Healthcare, Milwaukee, WI, US).

### 2.2 TTVI

Technical aspects of the tricuspid TEER [19] as well as of the heterotopic caval valve implantation using the TricValve device [20] have already been described. The device was selected on an individual basis by an interdisciplinary board of interventional cardiologists and cardiac surgeons based on the patient's comorbidities, underlying cardiac pathology, and individual anatomical conditions.

All patients were under general anesthesia during the procedure, and fluoroscopic and continuous TOE echocardiographic guidance were used for tricuspid TEER. Heterotopic caval valve implantation with the TricValve device (P&F Products&Features, Vienna, Austria) was accompanied by fluoroscopy and TTE echocardiography. Right atrial pressure and v‐wave were measured during the procedure. Concerning TEER one to two devices using the PASCAL system (Edwards Lifesciences, Irvine, USA) or TriClip device (Abbott Vascular, Menlo Park, USA) had been implanted during one procedure in order to achieve a significant reduction of TR.

### 2.3 Ultrasound Evaluation of VVC

The abdominal ultrasound was performed systematically following a standardized protocol to ensure consistent and reproducible results. All patients completed the standardized protocol. The sonographer was systemically blinded to the clinical context, the echocardiographic findings, the procedural success, or follow‐up status.

Organ sizes of the spleen and kidneys were measured using the rotational ellipsoid formula. Volume status included pleural effusions, ascites, and IVC diameter with sniff‐test for respiratory variability.

The estimation of the VVC included Doppler measurements of the splenic artery and splenic vein at the hilus, the renal artery and vein at the corticomedullary junction, the liver artery, the portal vein at the liver hilus, and the right liver vein. The blood flow profiles of spleen, kidneys and liver were rated qualitatively by a specially created rating scale (Figure 1A–C). In a prior study [21], we described 5 grades of renal vein and portal vein blood flow patterns. RVFP I° represents the regular continuous flow, RVFP II° represents discontinuous pulsatile flow, RVFP III° indicates discontinuous biphasic flow, RVFP IV° shows a discontinuous monophasic flow, and RVFP V° corresponds to pendular (oscillating) flow within the renal veins and represents the maximum possible VVC. These patterns could be seen according to Figure 1. The images of all renal vein flow profiles were assessed independently by two readers using the rating scale, showing excellent values for the interobserver reproducibility analysis (Cohen's kappa 0.976).

**Figure 1:**
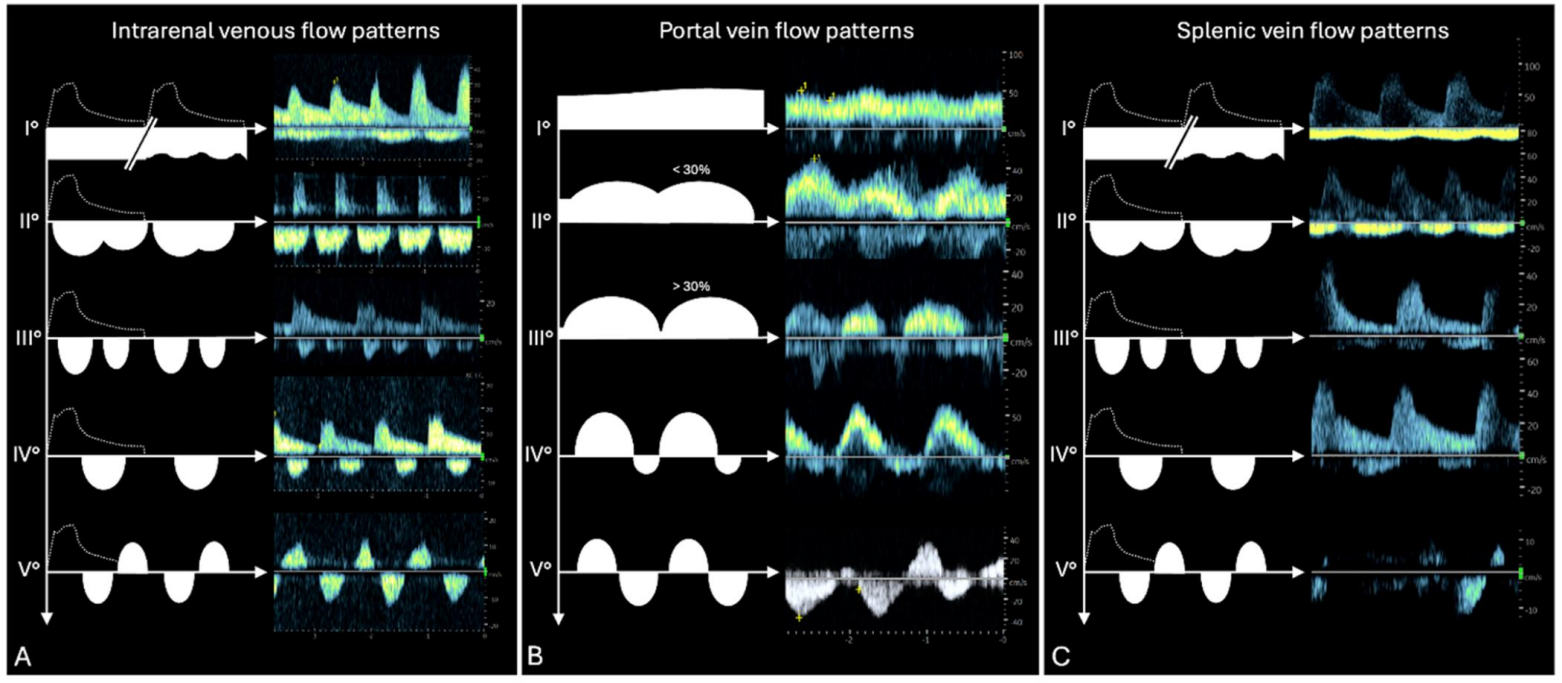
Rating scales to determine the visceral venous congestion in the kidneys, liver and spleen. 5 grade rating scale describing intrarenal venous flow patterns (A), portal vein flow profiles (B) and splenic vein flow profiles (C).

After the qualitative analysis, the percentage of renal venous flow (VFI) was calculated using the venous flow duration and the cardiac cycle time using the following formula and Figure 2.

**Figure 2:**
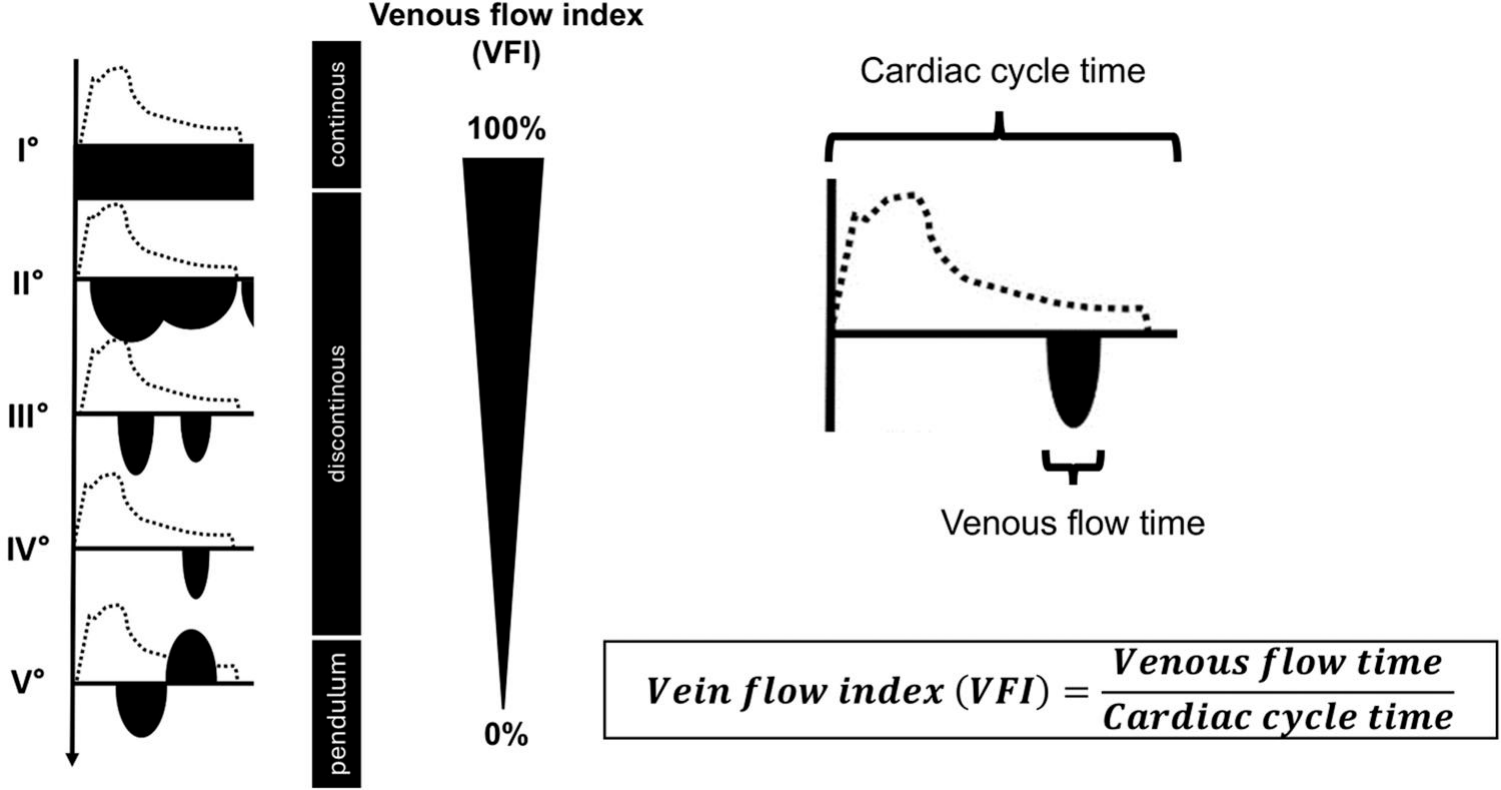
Description of the vein flow index (VFI). The VFI describes the percentage of the duration of the venous flow duration and the cardiac cycle time. Dotted lines represent the arterial flow profile of the kidney, the negative lines represent the venous blood flow. A typical, continuous venous blood flow pattern grade I° has a VFI of 100%. Grade V° represents a VFI of 0%, because there is no effective, blood movement within the renal vein due to the pendulum blood.

$\mathrm{Venous}\;\mathrm{flow}\;\mathrm{index}\;\left(\mathrm{VFI}\right)\;=\;\frac{\mathrm{Venous}\;\mathrm{flow}\;\mathrm{time}}{\mathrm{Cardiac}\;\mathrm{cycle}\;\mathrm{time}}$

We prioritized the renal venous flow index (VFI) over the venous stasis index (VSI), as it provides more physiologically relevant information on visceral congestion. Sonographic and echocardiographic parameters were available for all patients at all time points.

### 2.4 Statistical Analysis

The prespecified primary endpoint was the change in worst‐kidney RVFP grade IV–V between baseline and follow‐up in the TEER cohort. Secondary endpoints included changes in renal VFI, portal vein flow patterns, VExUS score, and clinical parameters such as NYHA class, 6‐min walk distance, and diuretic dose. Given the pilot, hypothesis‐generating nature of this study and the limited sample size, additional analyses were exploratory and should be interpreted descriptively rather than as confirmatory statistical evidence.

Continuous variables are reported as mean ± standard deviation when normally distributed, continuous variables with skewed distribution as median with interquartile range [first quartile‐third quartile]. Categorical variables are presented in numbers and percentages. Differences between groups were assessed using Student's *t*‐test for normally distributed data, Mann−Whitney‐*U* test for ordinal or skewed data, Pearson's chi‐squared test for nominal data, and Fisher's exact test for dichotomous data. Differences in paired samples were tested using paired t‐tests for normally distributed data, Wilcoxon signed‐rank tests for skewed or ordinal data, and McNemar's test for dichotomous data. To assess differences in repeated measures between two independent groups, Mixed Analysis of Variance was used. To evaluate correlation between ordinal variables, Spearman's rank correlation coefficient was calculated. A two‐sided *p* value of <0.05 was considered statistically significant. Statistical analyses were performed using SPSS Statistics 29 (IBM, Armonk, USA) and GraphPad Prism 10 (Dotmatics, Boston, USA).

## 3 Results

### 3.1 Baseline Characteristics

Baseline characteristics are shown in Table 1. TEER using PASCAL (*n* = 21) or TriClip (*n* = 3) was performed in 24 patients, and 6 received heterotopic caval valve implantation. Most patients (66.7%) were NYHA class III. Mean age was 77.8 ± 4.1 years. The majority of patients were female (*n* = 20, 66.7%). All patients suffered from atrial fibrillation.

**Table 1:** Baseline characteristics of the study population—RVFP I° versus RVFP II°−V°.

	All (*n* = 30)	RVFP I°(*n* = 7)	RVFP II°–V° (*n* = 23)	*p* value
Age	77.8 ± 4.1	79.6 ± 5.0	77.3 ± 3.8	0.201
Female gender	20 (66.7)	5 (71.4)	15 (65.2)	1.000
BMI, kg/m^2^	25.1 ± 4.2	24.7 ± 2.5	25.2 ± 4.6	0.766
CAD	13 (43.3)	4 (57.1)	9 (39.1)	0.666
Atrial fibrillation	30 (100.0)	7 (100.0)	23 (100.0)	−
Arterial hypertension	20 (66.7)	4 (57.1)	16 (69.6)	0.657
Diabetes mellitus	8 (26.7)	2 (28.6)	6 (26.1)	1.000
CKD (eGFR < 60 mL/min/1.73 m^2^)	23 (76.7)	6 (85.7)	17 (73.9)	1.000
Pacemaker	5 (16.7)	1 (14.3)	4 (17.4)	1.000
Loop diuretic dose, mg furosemide equivalent^a^	60.0 [20.0–120.0]	55.0 [20.0–80.0]	60 [20–120 mg/d]	0.485
NYHA functional class				0.764
II	10 (33.3)	2 (28.6)	8 (34.8)	
III	20 (66.7)	5 (71.4)	15 (65.2)	
6MWT, m	255.8 ± 93.2	276.0 ± 118.8	250.2 ± 88.2	0.595
TR grade				0.116
I	0	0	0	
II	0	0	0	
III	1 (3.3)	1 (14.3)	0	
IV	19 (63.3)	5 (71.4)	14 (60.9)	
V	10 (33.3)	1 (14.3)	9 (39.1)	
Type of intervention				0.290
TEER	24 (80.0)	7 (100.0)	17 (73.9)	
No. of implanted devices				0.854
1	11 (45.8)	3 (42.9)	8 (47.1)	
2	13 (54.2)	4 (57.1)	9 (52.9)	
Device type				1.000
TriClip	3 (10.0)	1 (14.3)	2 (8.7)	
PASCAL	21 (70.0)	6 (85.7)	15 (65.2)	
Heterotopic caval valve implantation	6 (20.0)	0	6 (26.1)	

^a^ 10 mg torasemide was converted to 20 mg furosemide equivalent.

The study population was divided into two groups: patients with normal intrarenal venous flow profiles (RVFP I°, *n* = 7) and patients with abnormal intrarenal venous flow profiles (RVFP II°−V°, *n* = 23). No significant differences were observed concerning age, chronic kidney disease (eGFR at baseline: 45.5 ± 19.6 mL/min/1.73 m^2^) or degree of TR. Most patients suffered from TR grade IV or V. 39.1% of patients with RVFP II°‐V° suffered from TR grade V, while TR grade V was only present in 14.3% of patients with RVFP I°.

Patients with RVFP I° had lower mean pulmonary artery pressures than RVFP II°−V° (20.1 ± 4.1 vs. 25.8 ± 4.2 mmHg, *p* = 0.005) and lower right atrial pressure (5.1 ± 2.3 vs. 11.7 ± 5.7 mmHg, *p* = 0.007) (Supporting Information S1: 2). The diameter of the IVC was narrower in patients with RVFP I° (1.83 ± 0.46 cm vs. 2.62 ± 0.65 cm, *p* = 0.009). Tricuspid vena contracta (VC) width was larger in patients with RVFP II°‐V° than in patients with RVFP I° reflecting an advanced extent of TR (9.7 ± 2.3 mm vs. 14.0 ± 4.6 mm, *p* = 0.027). No significant differences were observed concerning right ventricular function. However, there was a trend towards a larger right ventricular end‐diastolic diameter (RVEDD) in patients with RVFP II°−V° compared to patients with normal RVFP (RVEDD 48.7 ± 8.0 mm vs. 41.7 ± 9.0 mm, *p* = 0.058).

Patients with higher grades of RVFP showed significantly more abnormal portal and splenic vein flow patterns, indicating a greater degree of VVC prior to tricuspid intervention. This was further reflected by a lower relative venous flow time index (VFI) in the renal veins and an increased diameter of the left renal vein, representing a higher grade of venous visceral congestion. Additionally, patients with higher‐grade RVFP had more frequent findings of ascites and pleural effusion compared to those with RVFP I° (Table 3).

Because TEER and heterotopic caval valve implantation differ fundamentally in mechanism, patient selection, and disease severity, results are presented separately for the TEER cohort and for the smaller TricValve subgroup, which is described descriptively as an exploratory, more advanced end‐stage population (Table 2).

**Table 2:** Sonographic parameters of the TEER‐ and TricValve‐subgroup.

		TEER (*n* = 24)			TricValve (*n* = 6)	
	Pre	1 month	*p* value	Pre	1 month	*p* value
Weight	70.1 ± 11.7	69.5 ± 11.8	0.255	72.5 ± 11.6	70.6 ± 11.9	0.478
Renal volume						
left (mL)	158.3 ± 41.1	148.1 ± 44.1	0.174	128.5 ± 37.9	149.7 ± 24.8	0.111
Right (mL)	137.5 ± 44.1	129.1 ± 41.8	0.224	127.2 ± 31.8	126.6 ± 32.2	0.961
Total (mL)	295.8 ± 79.7	277.2 ± 78.6	0.104	256.1 ± 62.0	276.3 ± 52.5	0.317
A. interlobares sinistra						
vMax (cm/s)	34.9 ± 14.6	31.1 ± 10.3	0.254	22.8 ± 6.3	32.5 ± 15.9	0.173
Resistive index (RI)	0.73 ± 0.06	0.69 ± 0.16	0.328	0.69 ± 0.04	0.71 ± 0.07	0.513
V. interlobares sinistra						
vMax (cm/s)	11.2 ± 4.1	10.4 ± 5.0	0.575	13.3 ± 4.6	8.1 ± 4.8	0.213
Relative venous flow time index (VFI)	0.66 ± 0.37	0.81 ± 0.32	0.082	0.15 ± 0.19	0.42 ± 0.34	0.076
Venous stasis index (VSI)	0.34 ± 0.37	0.19 ± 0.32	0.082	0.82 ± 0.23	0.45 ± 0.32	0.089
A. interlobares dextra						
vMax (cm/s)	31.0 ± 10.1	31.2 ± 7.5	0.932	24.0 ± 5.7	28.7 ± 13.3	0.420
Resistive index (RI)	0.72 ± 0.06	0.72 ± 0.06	0.871	0.70 ± 0.05	0.67 ± 0.06	0.765
V. interlobares dextra						
vMax (cm/s)	10.4 ± 2.8	10.5 ± 4.5	0.970	11.5 ± 2.2	12.4 ± 5.6	0.799
Relative venous flow time index (VFI)	0.57 ± 0.40	0.81 ± 0.32	**0.014**	0.12 ± 0.14	0.50 ± 0.46	0.052
Venous stasis index (VSI)	0.43 ± 0.40	0.19 ± 0.32	**0.014**	0.91 ± 0.14	0.50 ± 0.46	**0.034**
V. renalis sinister						
Diameter max. (mm)	0.7 ± 0.6	0.3 ± 0.4	0.056	0.9 ± 0.2	0.7 ± 0.3	0.222
Diameter min. (mm)	0.3 ± 0.4	0.2 ± 0.3	0.260	0.8 ± 0.2	0.6 ± 0.3	0.198
Ratio Diameter (max./min.)	2.1 ± 1.7	1.5 ± 1.0	0.120	1.1 ± 0,1	1.2 ± 0.2	0.347
Vena cava inferior						
Diameter max. (cm)	2.4 ± 0.7	1.9 ± 0.8	**0.048**	2.6 ± 0.4	1.7 ± 1.3	0.190
Diameter min. (cm)	2.0 ± 0.8	1.6 ± 0.9	0.139	2.5 ± 0.4	1.6 ± 1.3	0.215
Ratio diameter (max./min.)	1.3 ± 0.4	1.3 ± 0.4	0.947	1.1 ± 0.1	1.0 ± 0.1	0.180
Portal vein						
vMax (cm/s)	16.6 ± 11.2	26.2 ± 6.6	0.053	33.0 ± 6.8	31.1 ± 6.7	0.310
vMin (cm/s)	16.6 ± 11.2	16.8 ± 6.6	0.940	5.3 ± 15.3	7.3 ± 20.3	0.867
Venous impedance index (VII)	0.5 ± 0.3	0.4 ± 0.2	0.568	1.1 ± 0.3	0.5 ± 0.1	**0.002**
A. hepatica						
vMax (cm/s)	53.0 ± 22.1	49.3 ± 24.4	0.447	52.1 ± 34.4	44.4 ± 8.9	0.658
Resistive index (RI)	0.7 ± 0.1	0.7 ± 0.2	0.173	0.7 ± 0.1	0.7 ± 0.1	0.492
Pleural effusion left	0	0	−	2 (33.3)	2 (33.3)	1.000
Pleural effusion right	2 (8.3)	3 (12.5)	1.000	1 (16.7)	2 (33.3)	1.000
Ascites	1 (4.2)	0	1.000	4 (66.7)	2 (33.3)	0.500
RVFP patterns left			**0.010**			0.059
I°	8 (33.3)	15 (62.5)		0	1 (16.7)	
II°	1 (4.2)	0		0	0	
III°	1 (4.2)	2 (8.3)		0	0	
IV°	10 (41.7)	5 (20.8)		2 (33.3)	4 (16.7)	
V°	4 (16.7)	2 (8.3)		4 (66.7)	1 (16.7)	
RVFP patterns right			**0.002**			0.066
I°	7 (29.2)	14 (58.3)		0	2 (33.3)	
II°	1 (4.2)	2 (8.3)		0	0	
III°	2 (8.3)	2 (8.3)		0	1 (16.7)	
IV°	8 (33.3)	4 (16.7)		2 (33.3)	1 (16.7)	
V°	6 (25.0)	2 (8.3)		4 (66.7)	2 (33.3)	
Liver vein flow patterns			0.579			0.317
I°	20 (83.3)	22 (91.7)		1 (16.7)	2 (33.3)	
V°	3 (12.5)	2 (8.3)		4 (66.7)	4 (66.7)	
Portal vein flow patterns			0.054			**0.043**
I°	15 (62.5)	19 (79.2)		0	3 (50.0)	
II°	7 (29.2)	5 (20.8)		0	2 (33.3)	
III°	0	0		0	0	
IV°	1 (4.2)	0		6 (100.0)	0	
V°	1 (4.2)	0		0	1 (16.7)	
Splenic vein flow patterns			**0.012**			0.066
I°	11 (45.8)	18 (75.0)		0	0	
II°	7 (29.2)	4 (16.7)		0	4 (66.7)	
III°	1 (4.2)	1 (4.2)		1 (16.7)	0	
IV°	4 (16.7)	1 (4.2)		2 (33.3)	1 (16.7)	
V°	1 (4.2)	0		3 (50.0)	0	
VExUS score			0.119			0.063
0	4 (16.7)	11 (45.8)		0	2 (33.3)	
1	1 (4.2)	7 (29.2)		0	0	
2	5 (20.8)	4 (16.7)		0	2 (33.3)	
3	3 (12.5)	1 (4.2)		6 (100.0)	2 (33.3)	

**Table 3:** Ultrasound characteristics of the study population—right RVFP I° versus RVFP II°−V°.

	All (*n* = 30)	RVFP I° (*n* = 7)	RVFP II°−V° (*n* = 23)	
Splenic vein flow patterns				**0.003**
I°	11 (36.7)	6 (85.7)	5 (21.7)	
II°	7 (23.3)	1 (14.3)	6 (26.1)	
III°	2 (6.7)	0	2 (8.7)	
IV°	6 (20.0)	0	6 (26.1)	
V°	4 (13.3)	0	4 (17.4)	
RVFP patterns left				**0.001**
I°	8 (26.7)	6 (85.7)	2 (8.7)	
II°	1 (3.3)	0	1 (4.3)	
III°	1 (3.3)	0	1 (4.3)	
IV°	12 (40.0)	1 (14.3)	11 (47.8)	
V°	8 (26.7)	0	8 (34.8)	
RVFP patterns right				**< 0.001**
I°	7 (23.3)	7 (100.0)	0	
II°	1 (3.3)	0	1 (4.3)	
III°	2 (6.7)	0	2 (8.7)	
IV°	10 (33.3)	0	10 (43.5)	
V°	10 (33.3)	0	10 (43.5)	
Portal vein flow patterns				**0.027**
I°	15 (50.0)	6 (85.7)	9 (39.1)	
II°	7 (23.3)	0	7 (30.4)	
III°	0	0	0	
IV°	7 (23.3)	0	7 (30.4)	
V°	1 (3.3)	0	1 (4.3)	
Renal volume (mL)				
Total	287.8 ± 77.2	280.8 ± 86.7	290.0 ± 76.1	0.789
Left	152.4 ± 41.6	156.0 ± 38.3	151.3 ± 43.3	0.798
Right	135.4 ± 41.7	124.8 ± 53.8	138.6 ± 38.1	0.450
A. interlobares sinistra				
vMax (cm/s)	32.4 ± 14.2	37.1 ± 19.3	31.0 ± 12.4	0.327
Resistance index (RI)	0.72 ± 0.07	0.75 ± 0.08	0.71 ± 0.06	0.217
V. interlobares sinistra				
vMax (cm/s)	11.6 ± 4.1	9.6 ± 3.2	12.2 ± 4.3	0.160
Relative venous flow time index	0.56 ± 0.40	0.97 ± 0.08	0.43 ± 0.37	**0.001**
Venous stasis index (VSI)	0.43 ± 0.40	0.03 ± 0.08	0.56 ± 0.38	**0.001**
V. renalis sinistra				
Max. diameter (mm)	0.7 ± 0.5	0.7 ± 0.9	0.7 ± 0.4	0.936
Min. diameter (mm)	0.4 ± 0.4	0.0 ± 0.0	0.6 ± 0.4	**0.002**
Ratio	1.7 ± 1.3	4.0 ± 2.7	1.4 ± 0.5	**< 0.001**
A. interlobares dextra				
vMax (cm/s)	29.8 ± 9.6	33.9 ± 12.3	28.6 ± 8.6	0.204
Resistance index (RI)	0.72 ± 0.06	0.71 ± 0.08	0.72 ± 0.05	0.662
V. interlobares dextra				
vMax (cm/s)	10.6 ± 2.7	7.8 ± 1.4	11.5 ± 2.4	**0.001**
Relative venous flow time index	0.48 ± 0.40	1.00 ± 0.00	0.32 ± 0.32	**< 0.001**
Venous stasis index (VSI)	0.53 ± 0.41	0.00 ± 0.00	0.69 ± 0.33	**< 0.001**
Vena cava inferior (cm, diameter)				
VCI max. (cm)	2.6 ± 0.8	1.8 ± 1.0	2.7 ± 0.7	0.118
VCI min. (cm)	2.3 ± 0.8	1.2 ± 1.1	2.4 ± 0.7	**0.042**
VCI ratio	1.2 ± 0.4	1.9 ± 0.9	1.1 ± 0.2	**0.004**
V. portae				
vMax (cm/s)	29.9 ± 8.0	30.7 ± 5.1	29.6 ± 8.8	0.744
vMin (cm/s)	14.3 ± 12.7	22.7 ± 7.1	11.8 ± 13.0	**0.044**
Venous impedance index (VVI)	0.6 ± 0.4	0.4 ± 0.3	0.7 ± 0.4	0.160
A. hepatica				
vMax (cm/s)	52.9 ± 23.9	53.3 ± 8.7	52.7 ± 27.2	0.956
Resistive index (RI)	0.7 ± 0.1	0.7 ± 0.1	0.7 ± 0.1	0.775
Ascites	5 (17.2)	0	5 (22.7)	
Pleural effusion left	2 (6.7)	0	2 (8.7)	
Pleural effusion right	3 (10.0)	0	3 (13.0)	

### 3.2 Procedural Outcomes

No differences were observed concerning the number or type of implanted devices during T‐TEER. There was no procedure‐associated morbidity or mortality. As shown in Figure 3A the number of patients suffering from TR grade III or IV could be reduced from 83% (*n* = 20) to 25% (*n* = 6) following T‐TEER (*p* < 0.001). No patient exhibited TR grade V at follow‐up after T‐TEER (baseline 17% (*n* = 4). At follow‐up right ventricular enddiastolic diameter (RVEDD) decreased, indicating right ventricular remodeling (RVEDD at baseline 47.1 ± 8.6 mm vs. RVEDD at follow‐up 43.9 ± 8.5 mm, *p* = 0.027). In the TEER cohort, procedural success defined as a reduction of TR by ≥2 grades was associated with a more pronounced improvement in worst‐kidney RVFP. Patients with successful TEER showed a greater shift from higher‐grade (IV–V) to lower‐grade venous flow patterns and a larger increase in renal VFI compared with those without such TR reduction, underscoring the close association between effective TR reduction and amelioration of renal venous congestion (Figure 5). After intervention, renal, portal, and splenic vein flow patterns improved significantly, and VExUS scores decreased significantly, indicating reduced VVC, serving as a global indicator.

**Figure 3:**
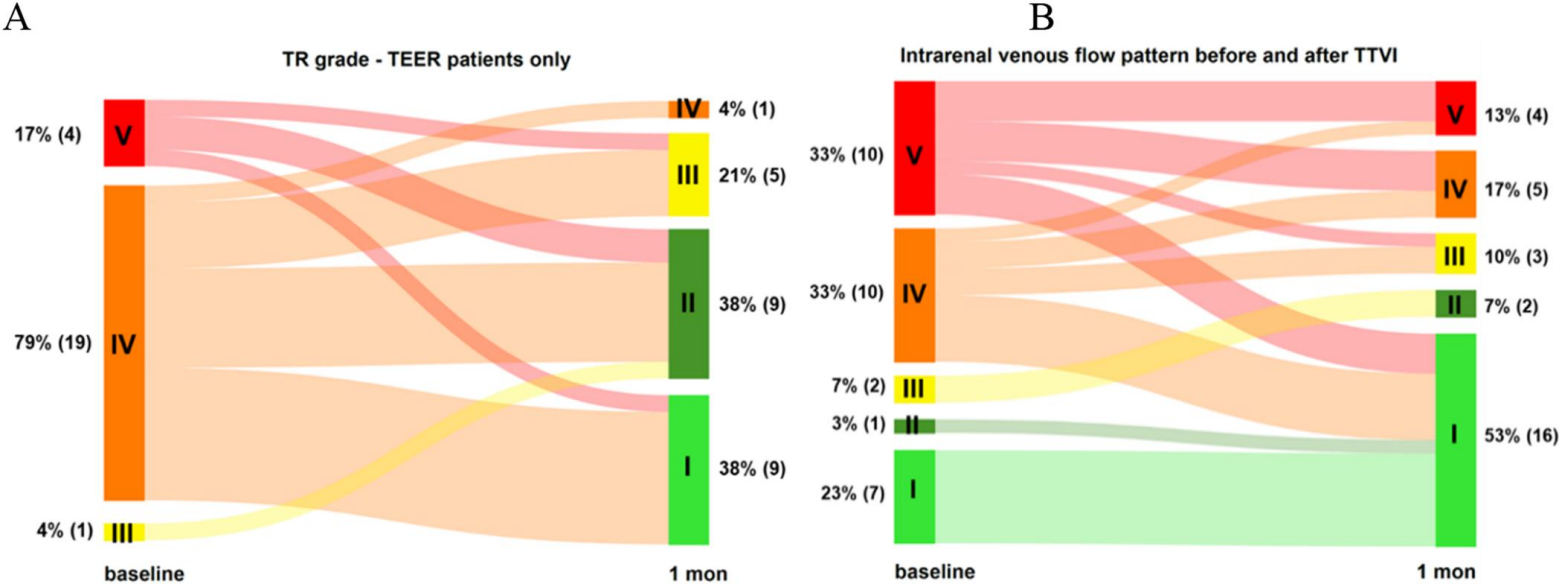
Change of the tricuspid regurgitation and the intrarenal flow pattern after tricuspid valve intervention. (A) Tricuspid regurgitation in TEER patients at baseline and follow‐up. (B) Intrarenal venous flow pattern at baseline and follow‐up.

As shown in Table 4 and Figure 3B, most patients demonstrated an improvement in renal vein flow within 1 month after tricuspid intervention. Prior to the intervention, 77% (23 patients) exhibited a renal vein flow profile greater than grade II°. This proportion decreased to 47% (14 patients) after the procedure. Severely impaired renal vein flow profiles (grades IV° and V°) decreased significantly from 66% (20 patients) before intervention to 30% (9 patients) afterward. 9 patients (30%) showed an improvement of ≥ 2 grades, 8 patients (27%) improved by 1 grade, 12 patients (40%) did not change the venous flow profile, and 1 patient (3.3%) deteriorated in the renal venous flow profile. The reduction of the renal venous flow profile correlated with the extent of the TR reduction (*p* = 0.041, Figure 5).

**Table 4:** Ultrasound characteristics of the study population at baseline and 1 month after tricuspid intervention.

	Baseline (*n* = 30)	1 month (*n* = 30)	*p* value
Splenic vein flow pattern			**0.002**
I°	11 (36.7)	18 (60.0)	
II°	7 (23.3)	8 (26.7)	
III°	2 (6.7)	1 (3.3)	
IV°	6 (20.0)	2 (6.7)	
V°	4 (13.3)	0	
RVFP patterns V. renalis sinistra			**0.002**
I°	8 (26.7)	16 (53.3)	
II°	1 (3.3)	0	
III°	1 (3.3)	2 (6.7)	
IV°	12 (40.0)	9 (30.0)	
V°	8 (26.7)	3 (10.0)	
RVFP patterns V. renalis dextra			**<0.001**
I°	7 (23.3)	16 (53.3)	
II°	1 (3.3)	2 (6.7)	
III°	2 (6.7)	3 (10.0)	
IV°	10 (33.3)	5 (16.7)	
V°	10 (33.3)	4 (13.3)	
Portal vein flow pattern			**0.006**
I°	15 (50.0)	22 (73.3)	
II°	7 (23.3)	7 (23.3)	
III°	0	0	
IV°	7 (23.3)	0	
V°	1 (3.3)	1 (3.3)	
VExUS‐score			**0.018**
0	4 (13.3)	13 (43.4)	
1	1 (3.3)	7 (23.3)	
2	5 (16.7)	6 (20.0)	
3	9 (30.0)	3 (10.0)	
Renal volume (mL)			
Total	287.8 ± 77.2	277.0 ± 73.3	0.279
Left	152.4 ± 41.6	148.4 ± 40.6	0.548
Right	135.4 ± 41.7	128.6 ± 39.6	0.254
A. interlobares sinistra			
vMax (cm/s)	32.5 ± 14.2	31.3 ± 11.3	0.714
Resistance Index (RI)	0.72 ± 0.07	0.70 ± 0.15	0.435
V. interlobares sinistra			
vMax (cm/s)	11.6 ± 4.1	10.0 ± 5.0	0.248
Relative venous flow time index (VFI)	0.56 ± 0.40	0.73 ± 0.35	**0.020**
Venous stasis index (VSI)	0.41 ± 0.39	0.22 ± 0.32	**0.022**
V. renalis sinistra			
Max. Diameter (mm)	0.7 ± 0.5	0.4 ± 0.4	**0.027**
Min. diameter (mm)	0.4 ± 0.4	0.3 ± 0.3	0.224
Ratio	1.8 ± 1.4	1.4 ± 0.8	0.144
A. interlobares dextra			
vMax (cm/s)	29.5 ± 0.97	30.7 ± 8.8	0.622
Resistance index (RI)	0.72 ± 0.06	0.72 ± 0.07	0.981
V. interlobares dextra			
vMax (cm/s)	10.6 ± 2.7	10.8 ± 4.6	0.855
Relative venous flow time index (VFI)	0.48 ± 0.40	0.74 ± 0.36	**0.002**
Venous stasis index (VSI)	0.53 ± 0.41	0.26 ± 0.36	**0.001**
Vena cava inferior (VCI, diameter)			
VCI max. (cm)	2.5 ± 0.6	1.8 ± 1.0	**0.020**
VCI min. (cm)	2.2 ± 0.7	1.6 ± 1.0	**0.045**
VCI ratio	1.3 ± 0.4	1.2 ± 0.3	0.882
V. portae			
vMax (cm/s)	29.9 ± 8.0	27.2 ± 6.8	**0.030**
vMin (cm/s)	14.3 ± 12.7	14.9 ± 11.0	0.850
Venous impedance index (VVI)	0.6 ± 0.4	0.5 ± 0.2	0.091
A. hepatica			
vMax (cm/s)	52.9 ± 23.9	48.4 ± 22.5	0.358
Resistive index (RI)	0.7 ± 0.1	0.7 ± 0.2	0.242
Ascites	5 (16.7)	2 (6.7)	0.250
Pleural effusion left	2 (6.7)	2 (6.7)	1.000
Pleural effusion right	3 (10.0)	5 (16.7)	0.687

These findings were also reflected in improved renal vein flow time index (VFI) and a reduction in the renal stasis index (VSI). Additionally, the diameter of the inferior vena cava decreased significantly.

T‐TEER patients showed significant improvement in the renal vein flow; heterotopic caval valve patients showed only a trend. Portal vein flow patterns indicated more pronounced congestion in patients with heterotopic caval valve implantation, which improved significantly after the intervention compared to T‐TEER patients. No significant differences in VExUS scores were observed between the two groups.

### 3.3 Clinical Outcomes

After tricuspid intervention, patients displayed a significant improvement in the NYHA‐class (26 patients, 86.7% showed NYHA class I‐II at follow‐up vs. 10 patients, 33.3% at baseline). Functional capacity improved after TTVI (6 min walk test 263.7 ± 95.9 m vs. 316.7 ± 63.6 m, *p* = 0.004).

Before tricuspid intervention, patients with RVFP II°−V° had a trend to higher doses of diuretics in comparison to patients with RVFP I°, but it showed no significance (60 mg/day [20−120] vs. 55.0 mg/day [20.0−80.0], *p* = 0.485).

At baseline there was no difference in the clinical signs of decompensation between both groups (RVFP I° *n* = 4; 57.1% vs. RVFP II°−V° *n* = 13; 56.5%, *p* = 0.977 Pearson‐Chi‐Quadrat‐Test) or the need of i.v. diuretics (RVFP I° *n* = 3; 42.9% vs. RVFP II°−V° *n* = 12; 52.2%, *p* = 0.666 Pearson‐Chi‐Quadrat‐Test).

In contrast, patients with RVFP I° at follow‐up (*n* = 16) had significantly lower doses of furosemide than patients with RVFP II°−V° (*n* = 14) (35.0 mg/day [20.0−40.0] vs. 60.0 mg/day [40.0–97.5] *p* = 0.035). When patients with an initial RVFP II°−V° showed an improvement of ≥ 1 grade (*n* = 17), they had significantly lower doses of furosemide than patients without improvement (40.0 mg/day [30.0−50.0] vs. 70.0 mg/day [50.0−400.0], *p* = 0.009).

## 4 Laboratory Results

Laboratory results of the study population are shown in Supporting Information S1: 3. There were no significant differences between both groups especially concerning NTproBNP (baseline: 2042.0 pg/mL [1117.0−4017.25] vs. follow‐up: 2531.5 pg/mL [1336.5−4400.8], *p* = 0.071) and GFR (baseline: 45.5 ± 19.6 ml/min/1.73 m^2^ vs. follow‐up: 45.2 ± 19.2 mL/min/1.73 m^2^, *p* = 0.846). After tricuspid intervention, there was a significant reduction of bilirubin (0.96 ± 0.43 vs. 0.76 ± 0.36, *p* = 0.001).

## 5 Discussion

Tricuspid valve regurgitation is a serious disorder that often leads to considerable life restrictions, particularly in advanced stages. TR is linked to a 65% rise in hospitalizations and death, with higher risk in patients with RV dysfunction [22].

Severe TR is characterized by the cardinal symptoms of VVC, which influence patients' prognosis even when mild [22]. Forado‐Benatar et al. described the prevalence of TR, right ventricular dysfunction, and renal congestion as a cardiorenal triangle [4]. The progression of TR is accompanied by right ventricular dysfunction, increased venous pressure, and a reduction of stroke volume. These alterations cause VVC and influence hemodynamics. In the long term, increased VVC leads to renal and liver dysfunction. Treatment options include decongestion as well as TTVI [4].

Isolated surgery for patients with severe TR is accompanied by a high in‐hospital mortality rate, therefore, the rates of singular TR surgery are low. Today TR can be treated by different transcatheter interventions. These methods are an alternative for high‐risk or inoperable patients according to current guidelines [23].

The prognostic value of a TTVI is currently unclear. Patients gain a higher level of quality of life after TEER, but there was no difference in hospitalizations and death in patients with tricuspid intervention compared to medical treatment [8]. Not all patients benefit equally from TTVI, for example, patients with renal insufficiency [8]. Patients with end‐stage right ventricular failure are also associated with a poor response to TR intervention [24].

Patient selection and timing of TTVI is challenging. Therefore, we hypothesized that not only the quantification of TR is essential for TTVI but also the hemodynamic consequences of TR that is, VVC resulting in progressive end‐organ failure, may be crucial for patient selection and timing.

In this study, we analysed the prevalence and potential change of VVC in patients undergoing TTVI. We developed a model of duplex parameters of renal veins and portal vein to describe the grade of VVC.

We were able to show that patients eligible for a TTVI start at different grades of VVC. This is interesting because the ideal timing of TR‐intervention is yet not clear, in early stages the symptoms are low, but the potential effect of TR‐intervention is high, due to the not yet existing end‐organ deterioration. In later stages – in patients with manifest end‐organ failure—a TR‐intervention does not lead to the same benefits as in other patients. Baseline end‐organ function was associated with HF hospitalization and death in patients with severe TR [14].

Our system provides a method to measure and grade the VVC and to identify patients at risk for irreversible organ dysfunction. Patients with TR suffered from a high degree of renal and hepatic congestion (77% patients with a RVFP ≥ II° and 27% of patients with a portal vein grade ≥ III°). We were able to determine this based on the venous flow profiles in the kidney and the flow pattern in the portal vein.

While normal RVFP without VVC shows a continuous venous flow pattern, the central sign of VVC is the occurrence of discontinuous renal venous flow, which represents an increased pressure in the venous system. In the most severe cases, patients exhibit pendulum flow in the renal vein, which corresponds to a net zero blood flow. The extent of renal vein flow correlates with the prognostically relevant RA pressure (RAP) and the VC.

After TTVI, patients responded differently to the treatment. Changes in the VVC occurred directly after the intervention and stayed in an optimal volume status to the end of the follow‐up. Most patients improved the grade of venous visceral congestion after TR intervention. Our results show that a reduction of TR leads to an improvement of visceral congestion, based on sonographic parameters (Figures 4 and 5). A higher reduction of TR leads to a higher rate of improvement of VVC. This is in line with a trend in the TRILUMINATE‐Study, where only patients with a higher rate of TR‐reduction do profit more in clinical outcomes, i.e. the MELD‐X‐Score [14].

**Figure 4:**
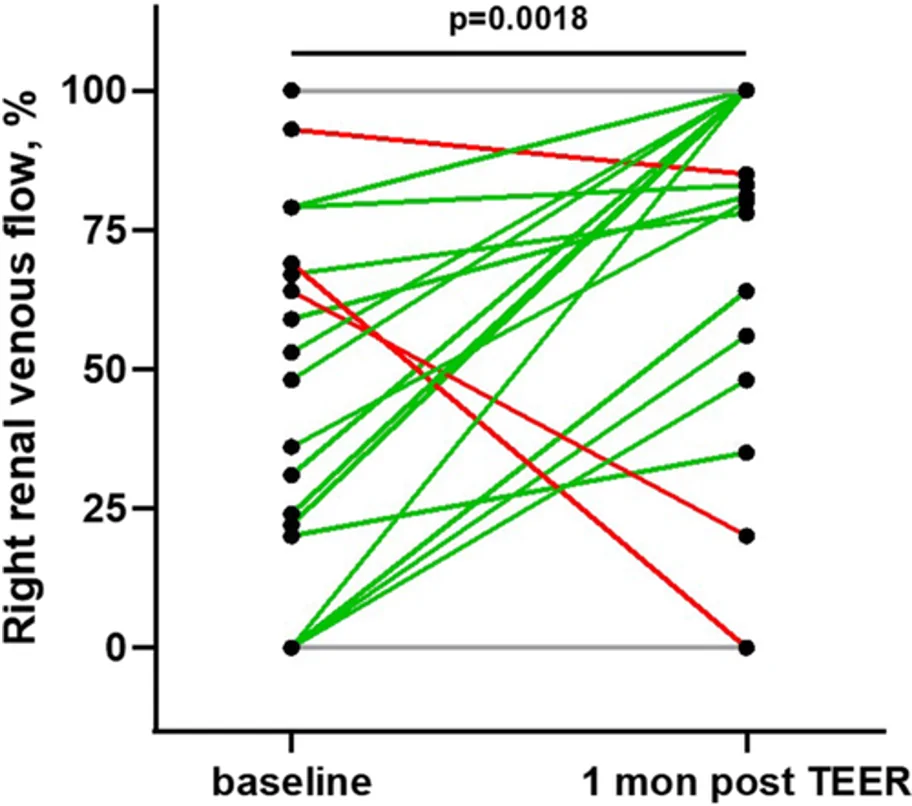
Change in the venous flow index of the right kidney after tricuspid intervention. The venous flow index represents the percentage of present blood flow in the renal vein in relation to the systolic duration (continuous blood flow = 100%, pendulum blood flow = 0%). The majority of patients showed an improvement of the renal venous flow time index after tricuspid intervention.

**Figure 5:**
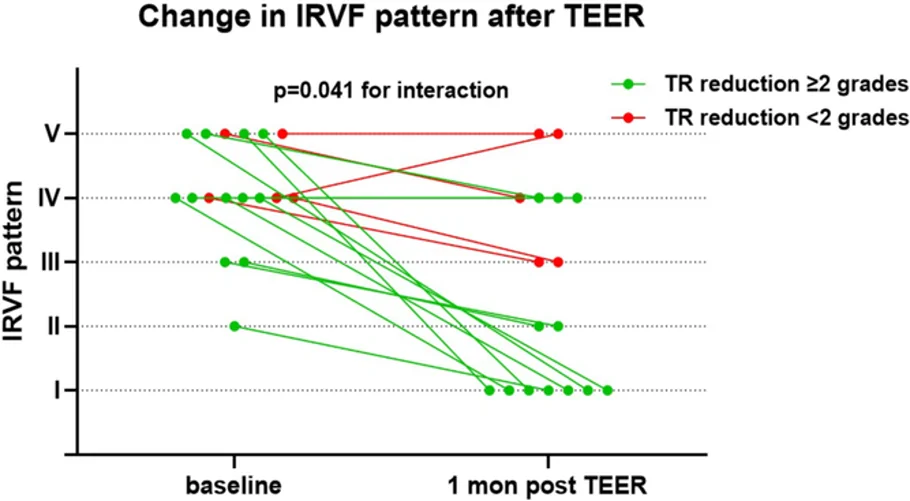
Change in renal vein flow profile after tricuspid valve intervention in correlation with the procedural success (TR reduction ≥ 2 grades). TR reduction ≥ 2 grades was associated with a significant higher reduction of the right renal vein flow pattern (green). The data were analyzed using a mixed‐design analysis of variance (mixed ANOVA. This interaction effect was considered statistically significant at the predefined significance level of *p* < 0.05.

The extent of the venous visceral congestion could be described even better by the VFI, which we introduced in this study. It represents the percentage of blood flow in the renal vein and therefore illustrating the blood flow through the kidneys. Overall, in our cohort the VFI prior to TTVI was 48% and improved to 74% after intervention. Further studies are needed regarding the long‐term‐course of venous congested patients in respect of the improvement of VVC and prognosis.

The main question arises whether the reduction of the VVC is the key‐success‐factor in TR‐interventions. Our study is the first one examining consistently VVC during TTVI. As prior studies (e.g., the TRILUMINATE trial) lack sonographic parameters to estimate the VVC, there is no possibility to compare our study collective with other studies in terms of the severity of VVC.

No improvement in kidney or liver function was seen in the short‐term course of this study. But this is in line with the findings of Jorde et al., which showed no renal or liver improvement in all TEER patients versus controls but displayed a positive signal in patients with interventional success [14]. It is worth considering in future studies whether patients with a higher grade of VVC and a sonographic improvement in the visceral congestion benefit more from TEER. Despite marked sonographic decongestion and a reduction in loop diuretic dose, renal function and NT‐proBNP did not improve over the short‐term follow‐up. This dissociation suggests that early changes in venous flow profiles and clinical status may precede or only partially translate into measurable changes in conventional laboratory markers. Alternatively, persistent neurohormonal activation and long‐standing end‐organ damage may limit the reversibility of eGFR and NT‐proBNP in this advanced TR population. Further, NT‐proBNP does not only reflect congestion but is also influenced by myocardial wall stress and heart failure. Longer‐term follow‐up and larger cohorts will be required to determine whether ultrasound‐based congestion improvement is associated with durable organ protection.

Renal insufficiency is a risk factor for hospitalization, morbidity and death in patients with TR [14]. Only 18% of patients with functionally relevant TR exhibit normal renal function, defined by an eGFR greater than 90 mL/min/1.73 m^2^ [11]. In addition to the elimination of toxins that are usually excreted in urine, impaired water removal and the subsequent water retention were particularly distressing for TR patients. It is evident that patients with severely impaired renal function are more prone to decompensation compared to other patients.

In our opinion, it remains uncertain if changes in the eGFR were the appropriate endpoint for determining the renal outcome. Although the elimination of urinary toxins in the kidney is still not impaired, patients suffer from volume overload. This could explain, why patients with VVC have a high rate of diuretic resistance related to the presence of tubular dysfunction and increased sodium avidity [25]. The pathophysiology shows that increased venous pressure leads to impaired water and sodium reabsorption and increased interstitial pressure within the kidney, leading to volume overload. Successful renal decongestion appears to be an effective method to improve the response to diuretics [4]. Instead of comparing the eGFR as the ultimate parameter for renal function, it is more meaningful to compare the amount of diuretics in these patients. In our cohort, a successful TEER procedure led to a significant decrease in the dose of diuretics. The dose of diuretics could be reduced even further when the RVFP improved ≥1 grade after the procedure. The most clinically relevant observation of our study is that effective reduction of severe TR by TEER was consistently associated with improvement in renal venous flow patterns, higher renal VFI, and lower VExUS scores. These findings support a mechanistic link between tricuspid intervention and amelioration of systemic venous congestion, while underscoring the need to further investigate whether this physiological improvement translates into long‐term clinical benefit.

This is the first time, where this finding was shown in a collective of patients undergoing TTVI.

To date there are different methods to measure the extend of VVC. We used a duplex sonographic system based on a five‐point rating scale of the vein flow within the renal vein, the portal vein, and the splenic vein. The use of the renal vein flow profile represents a simple approach to differentiate patients before TTVI. Because we did not find any patient with a higher rate of liver congestion without the presence of a high rate of renal congestion, we suggest that the degree of renal congestion is the critical issue. Actually, the most prominent diagnostic system is the VExUS score. This score is based on different blood flow patterns within the veins of kidney and liver. But this score is elaborate and has deficits in the identification of the highest rate of VVC, the venous pendulum flow. In our grading system, patients with severe VVC could be classified more easily.

There are limitations to this study. This study lacks a control group with patients with optimal conservative care that is, diuretic therapy. But this study was not designed to compare the effects of TTVI versus maximal conservative therapy. However, there is no recognized algorithm for the management of symptomatic TR patients before TEER [26]. This should be addressed in further studies, as the sonographic assessment of the VVC might be a beneficial marker to categorize patients.

Our findings demonstrate that ultrasound‐based assessment of renal and portal venous flow is feasible in this high‐risk TR population and that venous congestion profiles are responsive to successful TEER. However, in view of the small sample size, absence of a control group, and exploratory design, the data should be regarded as hypothesis‐generating rather than as validation of a tool for patient selection, optimal timing of intervention, or prediction of clinical benefit.

## 6 Conclusion

In summary, successful TEER in patients with severe TR was associated with amelioration of VVC as reflected by improved renal venous flow patterns, higher VFI, and lower VExUS scores. The proposed grading system and ultrasound protocol provide a practical framework for quantifying systemic venous congestion and its response to tricuspid intervention, but their role in patient selection, timing of intervention, and prediction of clinical benefit remains to be established. Future larger studies with extended follow‐up are needed to validate these physiological markers and determine whether they translate into durable organ protection and improved clinical outcomes.

## Author Contributions

Conceptualization: Franz Josef Putz, Christoph Birner, Kurt Debl, Michael Paulus, Christine Meindl; methodology: Franz Josef Putz, Christoph Birner, Bernhard Unsöld, Bernhard Banas, Lars S. Maier, Kurt Debl, Michael Paulus, Christine Meindl; formal analysis: Franz Josef Putz, Johannes Spahn, Michael Wester, Michael Paulus.; investigation: Franz Josef Putz, Johannes Spahn, Paul C. Kranert, Franziska Grewe, Moritz Haus, Michael Wester, Valeska Bienert, Maximilian Trum, Christian Schach, Lars S. Maier, Kurt Debl, Michael Paulus, Christine Meindl; data curation: Franz Josef Putz, Johannes Spahn, Michael Paulus, Christine Meindl; writing—original draft preparation: Franz Josef Putz, Johannes Spahn, Michael Paulus, Christine Meindl; writing—review and editing: Johannes Spahn, Paul C. Kranert, Franziska Grewe, Moritz Haus, Michael Wester, Valeska Bienert, Maximilian Trum, Christian Schach, Andreas Luchner, Christoph Birner, Bernhard Unsöld, Miriam C. Banas, Bernhard Banas, Lars S. Maier, Kurt Debl, Michael Paulus; visualization: Franz Josef Putz, Johannes Spahn, Michael Paulus, Christine Meindl.; supervision: Franz Josef Putz, Bernhard Banas, Lars S. Maier, Kurt Debl, Michael Paulus, Christine Meindl; project administration: Franz Josef Putz, Johannes Spahn, Michael Paulus, Christine Meindl.

All authors have read and agreed to the published version of the manuscript.

## Funding

The authors have nothing to report.

## Conflicts of Interest

M. Paulus and C. Meindl received speaker fees and travel support from Edwards Lifesciences.

## Artificial Intelligence

During the preparation of this work the authors did not apply any generative AI or AI‐assisted technologies in the writing process.

## Supporting information


Supporting File 1

Supporting File 2

## Data Availability

The data that support the findings of this study are available on request from the corresponding author. The data are not publicly available due to privacy or ethical restrictions.
