# Supplementary material for: Transcatheter Intervention Is Associated With Visceral Decongestion in Patients With Severe Tricuspid Regurgitation
Source: Clin Cardiol. 2026 Oct 22;49(10):e70497. doi: 10.1002/clc.70497 (PMC13688245; doi:10.1002/clc.70497)
Supplement: Supplementary file 1 [file CLC-49-e70497-s001.docx]

Supplement Material:

**Supplement 1: Correlation of the grade of intrarenal vein flow profile and the vena contracta and the right atrial pressure in patients with tricuspid regurgitation.**

**Supplement 2: Hemodynamic and echocardiographic characteristics of the study population – RVFP I° vs. RVFP II°-V°.**

|  | **All**  **(n=30)** | **RVFP I°**  **(n=7)** | **RVFP II°-V°**  **(n=23)** | **p-value** |
| --- | --- | --- | --- | --- |
| TR PISA Rvol, ml | 62.6 ± 26.0 | 44.8 ± 13.2 | 76.5 ± 26.7 | 0.057 |
| TR VC, mm | 13.0 ± 4.6 | 9.7 ± 2.3 | 14.0 ± 4.6 | **0.027** |
| sPAP, mmHg | 46.5 ± 16.7 | 40.9 ± 14.5 | 48.2 ± 17.2 | 0.317 |
| TAPSE/sPAP, mm/mmHg | 0.46 ± 0.39 | 0.51 ± 0.25 | 0.45 ± 0.32 | 0.594 |
| PAPm, mmHg | 24.4 ± 4.8 | 20.1 ± 4.1 | 25.8 ± 4.2 | **0.005** |
| PCWPm, mmHg | 17.5 ± 6.5 | 12.8 ± 5.0 | 18.6 ± 6.4 | 0.107 |
| RAPm, mmHg | 10.0 ± 5.8 | 5.1 ± 2.3 | 11.7 ± 5.7 | **0.007** |
| RAPv, mmHg | 15.8 ± 8.7 | 7.0 ± 2.6 | 17.6 ± 8.5 | 0.052 |
| RA area, cm² | 34.5 ± 10.8 | 31.0 ± 10.9 | 35.5 ± 10.7 | 0.339 |
| RVEDD, mm | 47.1 ± 8.6 | 41.7 ± 9.0 | 48.7 ± 8.0 | 0.058 |
| TAPSE, mm | 17.7 ± 2.9 | 18.4 ± 2.0 | 17.5 ± 3.1 | 0.473 |
| RV FAC, % | 40.0 ± 6.1 | 40.7 ± 4.0 | 39.7 ± 6.7 | 0.718 |
| E/e' | 12.5 ± 5.0 | 12.2 ± 5.2 | 12.6 ± 5.1 | 0.847 |
| VCI diameter, cm | 2.46 ± 0.69 | 1.83 ± 0.46 | 2.62 ± 0.65 | **0.009** |
| LVEF, % | 56.6 ± 7.6 | 59.7 ± 5.1 | 55.6 ± 8.1 | 0.216 |

**Supplement 3: Laboratory results of the study population at baseline and 1 month after tricuspid intervention.**

|  | **Baseline (n=30)** | **1 month (n=30)** | **p-value** |
| --- | --- | --- | --- |
| NTproBNP (pg/ml) | 2042.0 [1117.0-4017.25] | 2531.5 [1336.5-4400.8] | 0.071 |
| Creatinine (mg/dl) | 1.47 ± 0.65 | 1.48 ± 0.68 | 0.921 |
| eGFR (CKD-EPI, ml/min/1,73m²) | 45.5 ± 19.6 | 45.2 ± 19.2 | 0.846 |
| Total bilirubin (mg/dl) | 0.96 ± 0.43 | 0.76 ± 0.36 | **0.001** |
| Albumin (g/l) | 42.9 ± 4.7 | 43.1 ± 4.2 | 0.837 |
| GOT (U/l) | 29.8 ± 7.8 | 31.8 ± 11.5 | 0.197 |
| GPT (U/l) | 16.7 ± 4.7 | 19.6 ± 10.7 | 0.144 |
| GGT (U/l) | 110.9 ± 91.6 | 126.7 ± 102.6 | 0.111 |
