# Supplementary material for: Transcatheter Intervention Is Associated With Visceral Decongestion in Patients With Severe Tricuspid Regurgitation
Source: Clin Cardiol. 2026 Oct 22;49(10):e70497. doi: 10.1002/clc.70497 (PMC13688245; doi:10.1002/clc.70497)
Supplement: Supplementary file 2 [file CLC-49-e70497-s002.docx]

Supplement Material:

**Supplement 1: Correlation of the grade of intrarenal vein flow profile and the vena contracta and the right atrial pressure in patients with tricuspid regurgitation.**

**Supplement 2: Change in renal vein flow profile after tricuspid valve intervention in correlation with the procedural success (TR reduction >= 2 grades)**

**Supplement 3: Sonographic parameters of the TEER- and TricValve-subgroup**

|  |  | **TEER**  **(n=24)** |  |  | **TricValve**  **(n=6)** |  |
| --- | --- | --- | --- | --- | --- | --- |
|  | **pre** | **1 month** | **p-value** | **pre** | **1 month** | **p-value** |
| Weight | 70.1 ± 11.7 | 69.5 ± 11.8 | 0.255 | 72.5 ± 11.6 | 70.6 ± 11.9 | 0.478 |
| Renal volume |  |  |  |  |  |  |
| left (ml) | 158.3 ± 41.1 | 148.1 ± 44.1 | 0.174 | 128.5 ± 37.9 | 149.7 ± 24.8 | 0.111 |
| right (ml) | 137.5 ± 44.1 | 129.1 ± 41.8 | 0.224 | 127.2 ± 31.8 | 126.6 ± 32.2 | 0.961 |
| total (ml) | 295.8 ± 79.7 | 277.2 ± 78.6 | 0.104 | 256.1 ± 62.0 | 276.3 ± 52.5 | 0.317 |
| A. interlobares sinistra |  |  |  |  |  |  |
| vMax (cm/s) | 34.9 ± 14.6 | 31.1 ± 10.3 | 0.254 | 22.8 ± 6.3 | 32.5 ± 15.9 | 0.173 |
| Resistive Index (RI) | 0.73 ± 0.06 | 0.69 ± 0.16 | 0.328 | 0.69 ± 0.04 | 0.71 ± 0.07 | 0.513 |
| V. interlobares sinistra |  |  |  |  |  |  |
| vMax (cm/s) | 11.2 ± 4.1 | 10.4 ± 5.0 | 0.575 | 13.3 ± 4.6 | 8.1 ± 4.8 | 0.213 |
| Relative venous flow time index (VFI) | 0.66 ± 0.37 | 0.81 ± 0.32 | 0.082 | 0.15 ± 0.19 | 0.42 ± 0.34 | 0.076 |
| Venous stasis index (VSI) | 0.34 ± 0.37 | 0.19 ± 0.32 | 0.082 | 0.82 ± 0.23 | 0.45 ± 0.32 | 0.089 |
| A. interlobares dextra |  |  |  |  |  |  |
| vMax (cm/s) | 31.0 ± 10.1 | 31.2 ± 7.5 | 0.932 | 24.0 ± 5.7 | 28.7 ± 13.3 | 0.420 |
| Resistive Index (RI) | 0.72 ± 0.06 | 0.72 ± 0.06 | 0.871 | 0.70 ± 0.05 | 0.67 ± 0.06 | 0.765 |
| V. interlobares dextra |  |  |  |  |  |  |
| vMax (cm/s) | 10.4 ± 2.8 | 10.5 ± 4.5 | 0.970 | 11.5 ± 2.2 | 12.4 ± 5.6 | 0.799 |
| Relative venous flow time index (VFI) | 0.57 ± 0.40 | 0.81 ± 0.32 | **0.014** | 0.12 ± 0.14 | 0.50 ± 0.46 | 0.052 |
| Venous stasis index (VSI) | 0.43 ± 0.40 | 0.19 ± 0.32 | **0.014** | 0.91 ± 0.14 | 0.50 ± 0.46 | **0.034** |
| V. renalis sinister |  |  |  |  |  |  |
| Diameter max. (mm) | 0.7 ± 0.6 | 0.3 ± 0.4 | 0.056 | 0.9 ± 0.2 | 0.7 ± 0.3 | 0.222 |
| Diameter min. (mm) | 0.3 ± 0.4 | 0.2 ± 0.3 | 0.260 | 0.8 ± 0.2 | 0.6 ± 0.3 | 0.198 |
| Ratio Diameter (max./min.) | 2.1 ± 1.7 | 1.5 ± 1.0 | 0.120 | 1.1 ± 0,1 | 1.2 ± 0.2 | 0.347 |
| Vena cava inferior |  |  |  |  |  |  |
| Diameter max. (cm) | 2.4 ± 0.7 | 1.9 ± 0.8 | **0.048** | 2.6 ± 0.4 | 1.7 ± 1.3 | 0.190 |
| Diameter min. (cm) | 2.0 ± 0.8 | 1.6 ± 0.9 | 0.139 | 2.5 ± 0.4 | 1.6 ± 1.3 | 0.215 |
| Ratio diameter (max./min.) | 1.3 ± 0.4 | 1.3 ± 0.4 | 0.947 | 1.1 ± 0.1 | 1.0 ± 0.1 | 0.180 |
| Portal vein |  |  |  |  |  |  |
| vMax (cm/s) | 16.6 ± 11.2 | 26.2 ± 6.6 | 0.053 | 33.0 ± 6.8 | 31.1 ± 6.7 | 0.310 |
| vMin (cm/s) | 16.6 ± 11.2 | 16.8 ± 6.6 | 0.940 | 5.3 ± 15.3 | 7.3 ± 20.3 | 0.867 |
| Venous impedance index (VII) | 0.5 ± 0.3 | 0.4 ± 0.2 | 0.568 | 1.1 ± 0.3 | 0.5 ± 0.1 | **0.002** |
| A. hepatica |  |  |  |  |  |  |
| vMax (cm/s) | 53.0 ± 22.1 | 49.3 ± 24.4 | 0.447 | 52.1 ± 34.4 | 44.4 ± 8.9 | 0.658 |
| Resistive Index (RI) | 0.7 ± 0.1 | 0.7 ± 0.2 | 0.173 | 0.7 ± 0.1 | 0.7 ± 0.1 | 0.492 |
| Pleural effusion left | 0 | 0 | - | 2 (33.3) | 2 (33.3) | 1.000 |
| Pleural effusion right | 2 (8.3) | 3 (12.5) | 1.000 | 1 (16.7) | 2 (33.3) | 1.000 |
| Ascites | 1 (4.2) | 0 | 1.000 | 4 (66.7) | 2 (33.3) | 0.500 |
| RVFP patterns left |  |  | **0.010** |  |  | 0.059 |
| I° | 8 (33.3) | 15 (62.5) |  | 0 | 1 (16.7) |  |
| II° | 1 (4.2) | 0 |  | 0 | 0 |  |
| III° | 1 (4.2) | 2 (8.3) |  | 0 | 0 |  |
| IV° | 10 (41.7) | 5 (20.8) |  | 2 (33.3) | 4 (16.7) |  |
| V° | 4 (16.7) | 2 (8.3) |  | 4 (66.7) | 1 (16.7) |  |
| RVFP patterns right |  |  | **0.002** |  |  | 0.066 |
| I° | 7 (29.2) | 14 (58.3) |  | 0 | 2 (33.3) |  |
| II° | 1 (4.2) | 2 (8.3) |  | 0 | 0 |  |
| III° | 2 (8.3) | 2 (8.3) |  | 0 | 1 (16.7) |  |
| IV° | 8 (33.3) | 4 (16.7) |  | 2 (33.3) | 1 (16.7) |  |
| V° | 6 (25.0) | 2 (8.3) |  | 4 (66.7) | 2 (33.3) |  |
| Liver vein flow patterns |  |  | 0.579 |  |  | 0.317 |
| I° | 20 (83.3) | 22 (91.7) |  | 1 (16.7) | 2 (33.3) |  |
| V° | 3 (12.5) | 2 (8.3) |  | 4 (66.7) | 4 (66.7) |  |
| Portal vein flow patterns |  |  | 0.054 |  |  | **0.043** |
| I° | 15 (62.5) | 19 (79.2) |  | 0 | 3 (50.0) |  |
| II° | 7 (29.2) | 5 (20.8) |  | 0 | 2 (33.3) |  |
| III° | 0 | 0 |  | 0 | 0 |  |
| IV° | 1 (4.2) | 0 |  | 6 (100.0) | 0 |  |
| V° | 1 (4.2) | 0 |  | 0 | 1 (16.7) |  |
| Splenic vein flow patterns |  |  | **0.012** |  |  | 0.066 |
| I° | 11 (45.8) | 18 (75.0) |  | 0 | 0 |  |
| II° | 7 (29.2) | 4 (16.7) |  | 0 | 4 (66.7) |  |
| III° | 1 (4.2) | 1 (4.2) |  | 1 (16.7) | 0 |  |
| IV° | 4 (16.7) | 1 (4.2) |  | 2 (33.3) | 1 (16.7) |  |
| V° | 1 (4.2) | 0 |  | 3 (50.0) | 0 |  |
| VExUS Score |  |  | 0.119 |  |  | 0.063 |
| 0 | 4 (16.7) | 11 (45.8) |  | 0 | 2 (33.3) |  |
| 1 | 1 (4.2) | 7 (29.2) |  | 0 | 0 |  |
| 2 | 5 (20.8) | 4 (16.7) |  | 0 | 2 (33.3) |  |
| 3 | 3 (12.5) | 1 (4.2) |  | 6 (100.0) | 2 (33.3) |  |

**Supplement 4: Laboratory results of the study population at baseline and 1 month after tricuspid intervention.**

|  | **Baseline (n=30)** | **1 month (n=30)** | **p-value** |
| --- | --- | --- | --- |
| NTproBNP (pg/ml) | 2042.0 [1117.0-4017.25] | 2531.5 [1336.5-4400.8] | 0.071 |
| Creatinine (mg/dl) | 1.47 ± 0.65 | 1.48 ± 0.68 | 0.921 |
| eGFR (CKD-EPI, ml/min/1,73m²) | 45.5 ± 19.6 | 45.2 ± 19.2 | 0.846 |
| Total bilirubin (mg/dl) | 0.96 ± 0.43 | 0.76 ± 0.36 | **0.001** |
| Albumin (g/l) | 42.9 ± 4.7 | 43.1 ± 4.2 | 0.837 |
| GOT (U/l) | 29.8 ± 7.8 | 31.8 ± 11.5 | 0.197 |
| GPT (U/l) | 16.7 ± 4.7 | 19.6 ± 10.7 | 0.144 |
| GGT (U/l) | 110.9 ± 91.6 | 126.7 ± 102.6 | 0.111 |
